# Measurement error and reliability of three available 3D superimposition methods in growing patients

**DOI:** 10.1186/s13005-020-0215-7

**Published:** 2020-01-27

**Authors:** Cecilia Ponce-Garcia, Antonio Carlos de Oliveira Ruellas, Lucia Helena Soares Cevidanes, Carlos Flores-Mir, Jason P. Carey, Manuel Lagravere-Vich

**Affiliations:** 1 Division of Orthodontics, School of Dentistry, University of Alberta, Edmonton, Canada; 2 Faculty of Dentistry, Federal University of Rio de Janeiro, Rio de Janeiro, Brazil. Department of Orthodontics and Pediatric Dentistry, School of Dentistry, University of Michigan, Ann Arbor, USA; 3 Department of Orthodontics and Pediatric Dentistry, School of Dentistry, University of Michigan, Ann Arbor, USA; 4 Division of Orthodontics, School of Dentistry, University of Alberta, Edmonton, AB Canada; 5 Department of Mechanical Engineering, Faculty of Engineering, University of Alberta, Edmonton, AB Canada; 6 Division of Orthodontics, School of Dentistry, University of Alberta, Edmonton, AB Canada

**Keywords:** Three-dimensional superimposition, Cone beam computed tomography

## Abstract

**Introduction:**

Cone-Beam Computed Tomography (CBCT) images can be superimposed, allowing three-dimensional (3D) evaluation of craniofacial growth/treatment effects. Limitations of 3D superimposition techniques are related to imaging quality, software/hardware performance, reference areas chosen, and landmark points/volumes identification errors. The aims of this research are to determine/compare the intra-rater reliability generated by three 3D superimposition methods using CBCT images, and compare the changes observed in treated cases by these methods.

**Methods:**

Thirty-six growing individuals (11–14 years old) were selected from patients that received orthodontic treatment. Before and after treatment (average 24 months apart) CBCTs were analyzed using three superimposition methods. The superimposed scans with the two voxel-based methods were used to construct surface models and quantify differences using SlicerCMF software, while distances in the landmark-derived method were calculated using Excel. 3D linear measurements of the models superimposed with each method were then compared.

**Results:**

Repeated measurements with each method separately presented good to excellent intraclass correlation coefficient (ICC ≥ 0.825). ICC values were the lowest when comparing the landmark-based method and both voxel-based methods. Moderate to excellent agreement was observed when comparing the voxel-based methods against each other. The landmark-based method generated the highest measurement error.

**Conclusions:**

Findings indicate good to excellent intra-examiner reliability of the three 3D superimposition methods when assessed individually. However, when assessing reliability among the three methods, ICC demonstrated less powerful agreement. The measurements with two of the three methods (CMFreg/Slicer and Dolphin) showed similar mean differences; however, the accuracy of the results could not be determined.

## Introduction

Monitoring treatment progress and outcomes is pivotal to patient care [1]. Therefore, an important part of orthodontic treatment involves the study of longitudinal changes induced by growth and treatment in the dentofacial complex in individual patients [2–5]. Superimposing tracings of serial lateral cephalograms has facilitated knowledge about normal craniofacial growth and development as well as knowledge about the treatment effects produced by various orthodontic, orthopedic, and surgical procedures [3, 6]. A reference system is required for a superimposition to be able to determine exactly what and where changes occurred. Such references must be consistently visible in the cephalograms of the individual, and they must be stable within the time frame of the observation period [3, 7].

Several studies [8–14] have proposed the use of the anterior cranial base as reference for superimposition since there is little or no growth after 7–8 years of age when the spheno-ethmoidal synchondrosis ceases to grow. After that time a number of structures especially those associated with neural tissues remain stable and can be relied upon for superimposition [1].

Many types of superimposing methods have been used for 2D lateral cephalograms. However, 2D imaging does not fully represent a 3D structure, because much of the information is lost when 3D structures are depicted as 2D images [15–17]. Thus, while 2D cephalometric superimposition is the conventional method used to evaluate craniofacial growth and treatment outcomes, superimposition of CBCT scans, nowadays, allows a 3D visualization of these effects. Similar to cephalometric tracings, 3D models constructed from CBCT scans can be superimposed manually by registering common stable landmarks or by best fit of stable anatomical regions [18–20].

Three general methods of 3D cephalometric superimposition are well-published and used for clinical diagnosis and assessment of orthodontic treatment outcomes: (1) voxel-based, (2) point/landmark-based, and (3) surface-based. For overall superimposition, these methods use parts of the anterior cranial base, as a reference structure for CBCT superimposition, a structure known to have completed most of its growth before the adolescent growth spurt, therefore making it a quite stable reference structure for superimposition [14, 21].

Most of the limitations of 3D superimposition techniques are related to variability in imaging and landmark identification flaws and software/hardware related errors. In addition, most of the methods that have currently been proposed [22–25] for clinical settings are quite time-consuming. Thus, the establishment of a precise, reliable and efficient system to analyze images produced by 3D imaging is needed. Therefore, this study analyzed two voxel-based [CMFreg (Craniomaxillofacial registration) and Dolphin] and one point/landmark-based (LMD) superimposition methods. The voxel-based and the landmark-based methods have been previously validated, hence, this study evaluated and verified the reliability to measurement errors of the three methods when aligning the pre and post-growth/treatment images to provide clinicians with information about the reproducibility of the structural changes produced by growth and treatment effects in children and adolescents.

## Material and methods

A retrospective, observational longitudinal study was carried out on individuals that received comprehensive orthodontic treatment at the University of Alberta. Thirty-six patients with available pre- and post-treatment CBCTs were selected from a population of 11 to 14-year-old teenagers. The mean age of patients at the time of the initial CBCT was 12.4 ± 0.9 years (Cervical Vertebrae Maturation index [CVM] stage 3–4). The mean age at final CBCT was 14.3 ± 0.8 years. The sample included seventeen males and nineteen females.

The interval between pre-treatment (T1) and post-treatment (T2) ranged from 22 to 25 months apart. Fourteen patients presented Class I malocclusion, eight mild Class II malocclusion and fourteen mild Class III malocclusion. All patients received a non-extraction treatment and included rapid maxillary expansion, full fixed appliances, and intermaxillary elastics.

This study only analyzed previously gathered data from patients that participated in randomized clinical trials. No additional imaging was requested for these patients. Ethics approval was obtained by the Institutional Health Research Ethics Board at the University of Alberta for secondary data analysis.

CBCT volumetric data were taken using the iCAT New Generation Volumetric Scanner at 120 kV, 5 mA, and 8.9 s. Images were obtained and converted to Digital Imaging and Communications in Medicine (DICOM) format using the iCAT software with a voxel size of 0.3 mm.

Analysis of the images was carried out by one researcher using the respective superimposition techniques (CMFreg/Slicer, Dolphin and landmark-derived). Extensive training was required prior to superimposing with each method. Intra-observer reliability within each method was done using ten images and two repetitions each, with each measurement trial being at least 1 week apart. For the voxel-based methods reliability was tested twice, ten cases each, one performing a second superimposition with registration at the cranial base and one retracing landmarks only.

Reliability among the three methods was performed using the complete sample; the first trial of thirty-six cases of each method was used. Ten landmarks, used in previous studies [7, 23, 26–30], were marked on three-dimensional images at T1 and T2 with each of the three methods to assess reliability (Table 1).

**Table 1:** Landmark definition

Maxilla	
ANS	The tip of the bony anterior nasal spine, in the median plane
A-Point	The point at the deepest midline concavity on the maxilla between the anterior nasal spine and prosthion
PNS	The intersection of a continuation of the anterior wall of the pterygopalatine fossa and the floor of the nose, marking the dorsal limit of the maxilla
OrR	The lowest point in the inferior margin of the right orbit
OrL	The lowest point in the inferior margin of the left orbit
Mandible	
Me	Menton – The most inferior midline point on the mandibular symphysis
B-Point	The point at the deepest midline concavity on the mandibular symphysis between infradentale and pogonion
GoR	Constructed point of intersection of the right ramus and the mandibular plane
GoL	Constructed point of intersection of the left ramus and the mandibular plane
Pg	Pogonion – The most anterior of the bony chin in the median plane

### Voxel-based CMFreg/slicer method

This method uses two different open-source programs ITK-Snap (http://www.itksnap.org) and 3D Slicer (http://www.slicer.org). Using ITK-Snap software program (version 2.0.0) T1 and T2 DICOM files were opened and converted to GIPL (Guys Imaging Processing Lab) format for easy processing. Segmentations then were created using the GIPL.GZ files for both pre and post treatment scans using the 3D Slicer software program (version 4.7.0) to construct 3D volumetric label maps.

Then, surface models were created using the T1 segmentation in 3D Slicer to re-orient the head to establish a common coordinate system across subjects for group comparisons [31]. Once the head orientation step was completed, the T2 image was manually approximated in relation to T1 image using 3D Slicer. ITK-Snap was used to segment the area of the cranial base to be used as a reference for the superimposition using semi-automatic segmentation.

The registration (superimposition) of the T2 image upon the T1 image was carried out on the segmented cranial base, using the craniomaxillofacial tool and the setting growing rigid automatic registration in 3D Slicer. During the superimposition, T2 was reoriented guided by the best fit of the outlines of the anterior cranial base and automatically superimposed on a static T1, creating a registered T2 surface model.

**Fig. 1:**
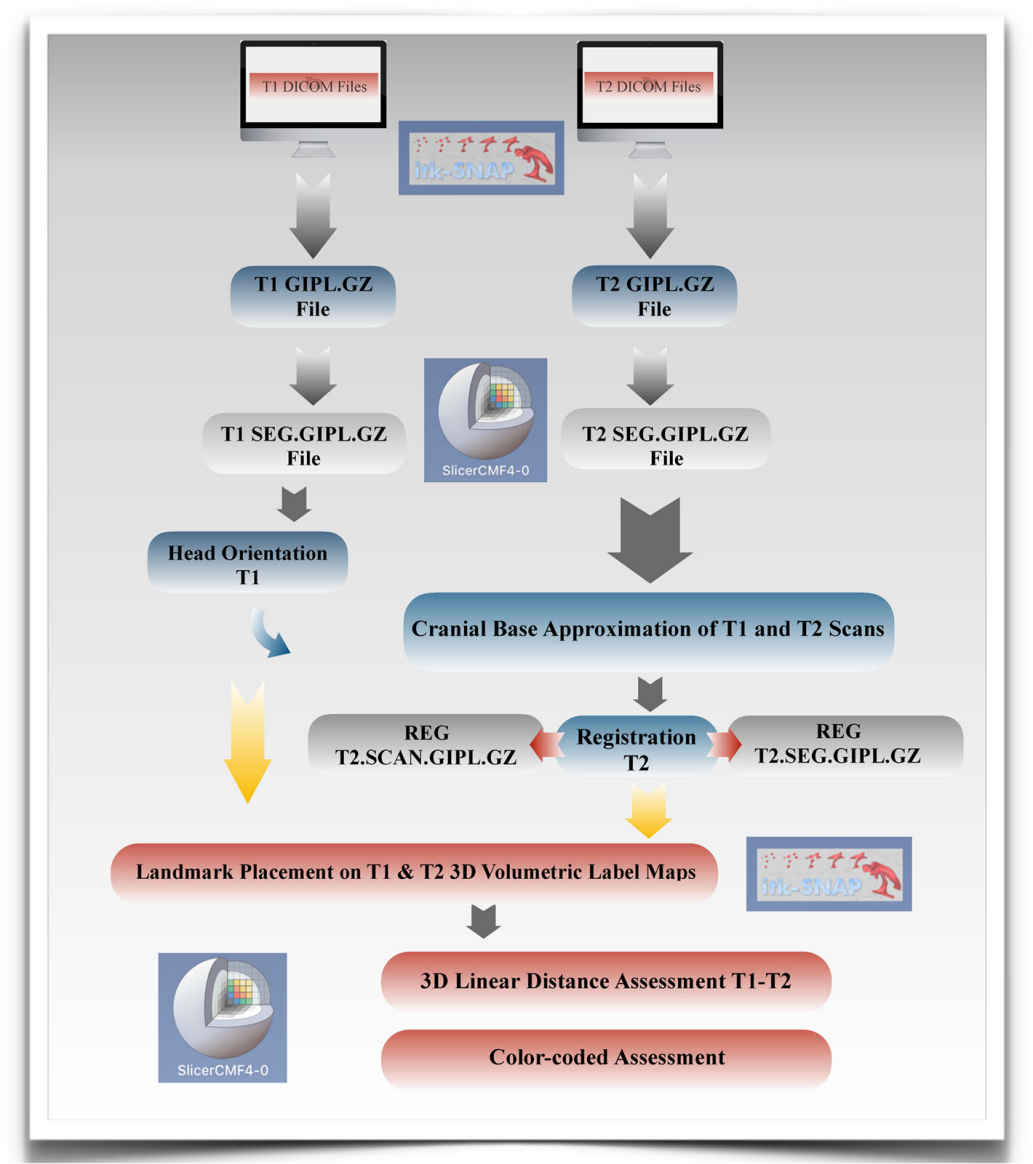
Flow Diagram CMFreg/slicer Method. This method uses two different programs ITK-Snap and 3D Slicer. T1 and T2 DICOM files are initially opened and converted to GIPL using ITK-Snap. Segmentations then are created using the GIPL.GZ files for both pre and post treatment scans using the 3D Slicer to construct 3D volumetric label maps. Surface models are created after using the T1 scan and segmentation in 3D Slicer to re-orient the head [1]. Once the T1 scan has been reoriented, the registration (superimposition) of the T2 image upon the T1 image is carried out on the segmented cranial base. Then T1 and T2 images are landmarked using ITK-Snap and new models are created to measure the absolute differences between the pre and post-treatment images

**Fig. 2:**
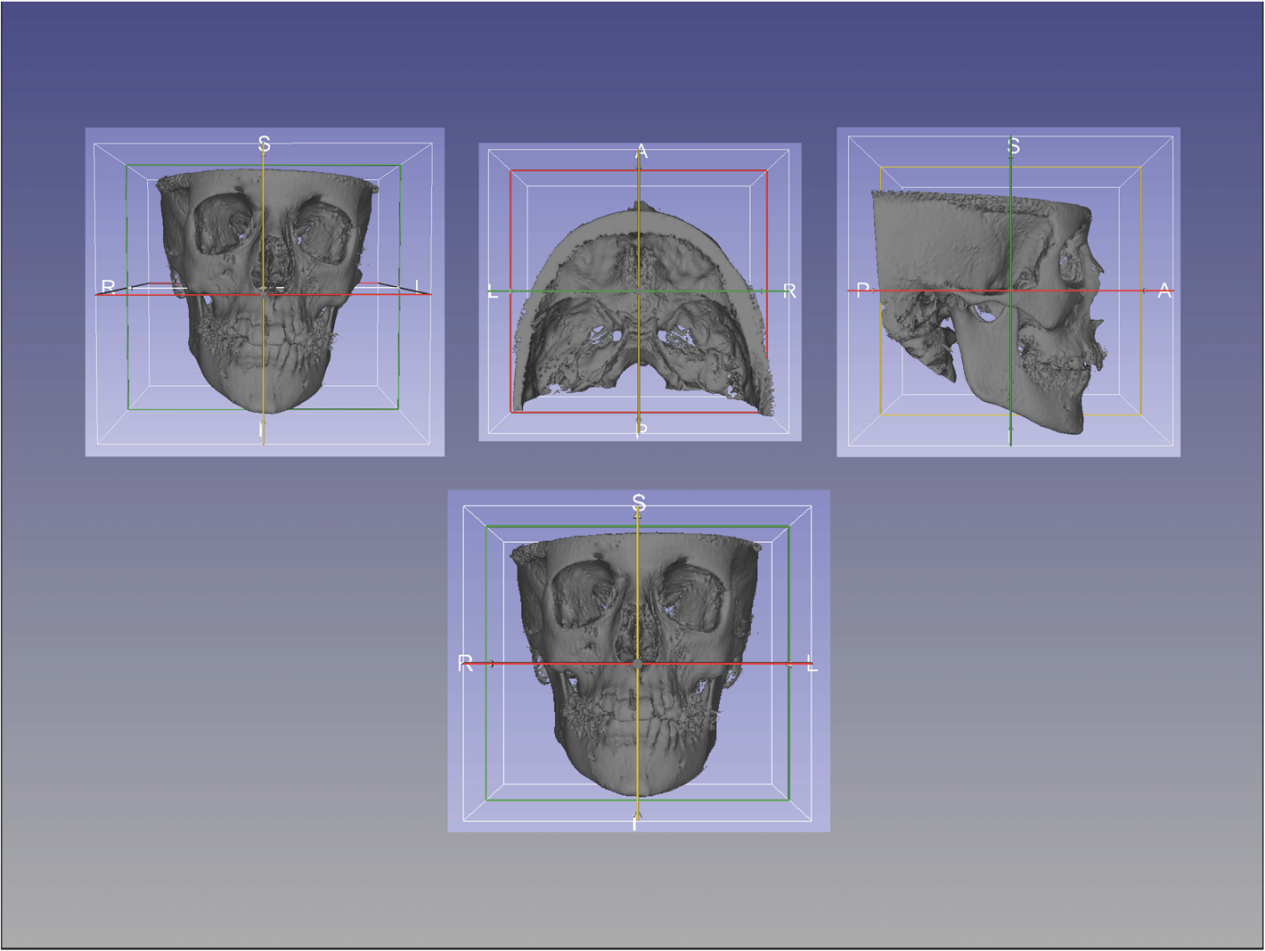
Head orientation with CMFreg/Slicer method. Using the Transform function pre and post-treatment images are reoriented utilizing Foramen Magnum, Crista Galli and Glabella on the sagittal plane, Frankfort horizontal (Porion-Orbitale) on the vertical plane, and Porion to Porion on the transverse plane

**Fig. 3:**
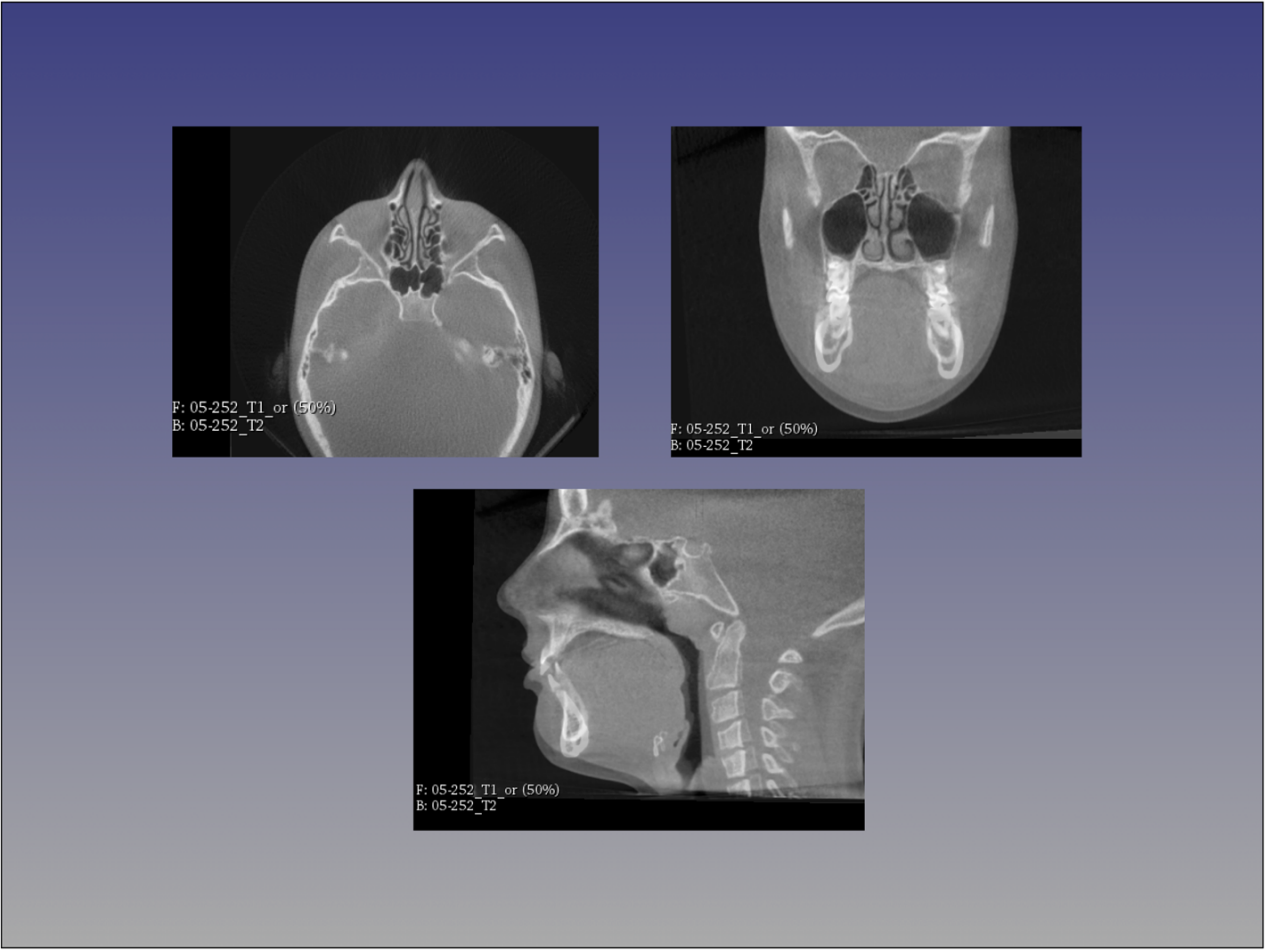
Cranial base approximation with CMFreg/Slicer method. Axial, coronal and sagittal views are used to superimpose pre and post images

**Fig. 4:**
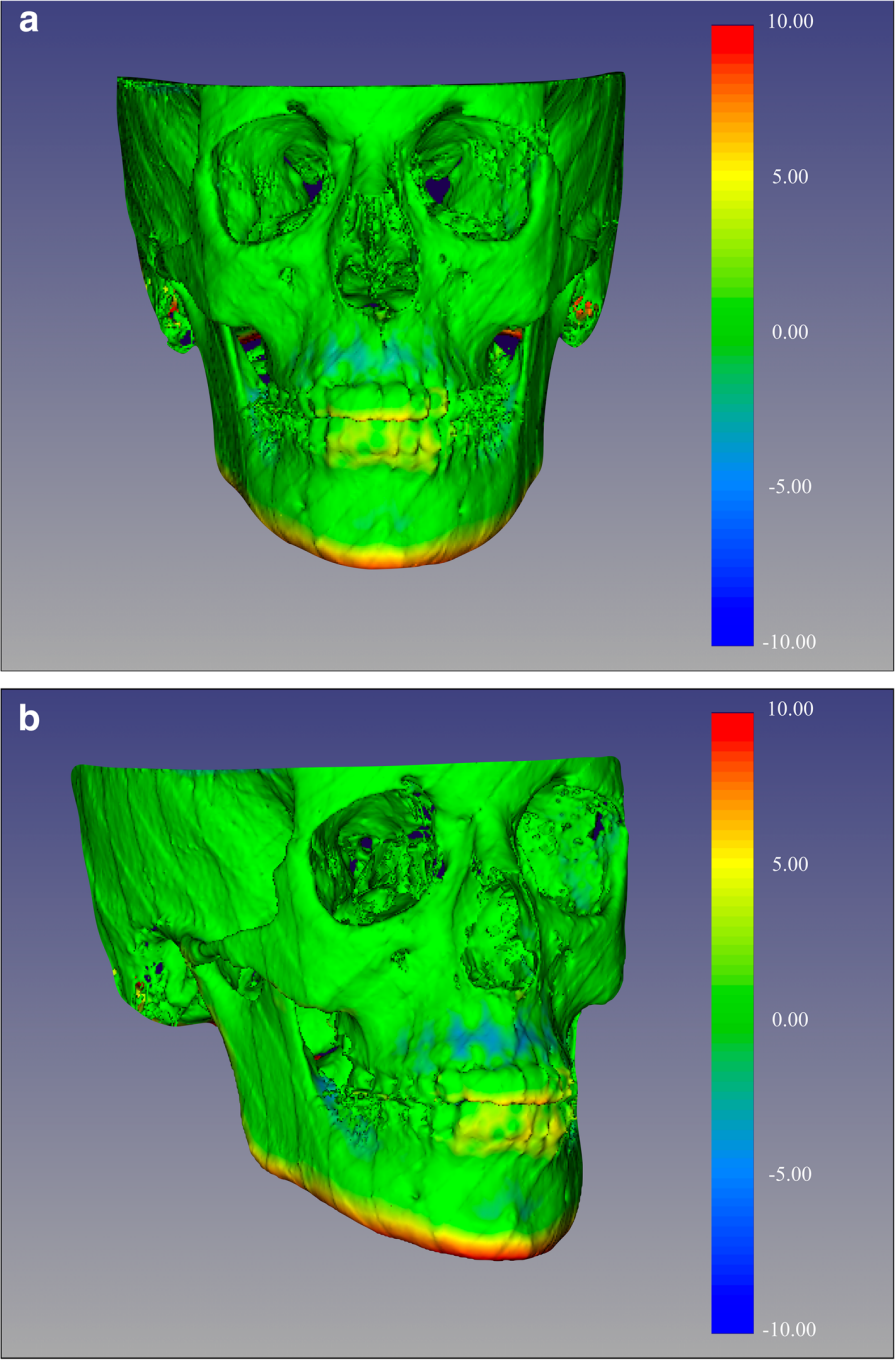
Color-coded map with CMFreg/Slicer method for visualization purposes only, not quantitative assessment. Frontal (Fig. 4a) and 45 degrees (Fig. 4b) views of the 3D color-coded maps showing the change in millimetres

Once the superimposition was completed, the T1 scan and segmentation, as well as the registered T2 scan and segmentation, were landmarked using ITK-Snap. Ten 3D landmarks were identified using the three views (axial, sagittal and coronal) for consistency of landmark location. After placing the defined landmarks to T1 and T2 images, 3D surface models were created using 3D Slicer. These models were utilized to measure the absolute differences between the pre and post-treatment images by applying the Q3DC module (Quantification in 3D and directional changes in each plane of the three planes of space). 3D linear distances between T1 and T2 of corresponding landmarks were quantified in the transversal (x-axis), antero-posterior (y-axis) and vertical (z-axis) direction (Figs. 1, 2, 3, and 4).

### Landmark-derived method

Using AVIZO software, the DICOM files were rendered into a volumetric image using 512 × 512 matrices giving a range of 400–420 DICOM slices. Sagittal, axial and coronal multiplanar slices, as well as the 3D image reconstructions, were used to determine the position of the seven landmarks used to superimpose the T1 and T2 images.

Given the coordinates of three reference landmarks for a plane, 3D visualization software can compute the plane; however, entering the three-point coordinates usually is a time-consuming repetitive manual process. A similar argument applies to determine the perpendicular distance. In order to resolve this issue, this study reproduced the mathematic procedure in Microsoft Excel. This allowed the reference planes and perpendicular distances to be automatically calculated whenever the landmark coordinates were updated.

Four landmarks were required to define a 3D anatomical reference co-ordinate system. The left and right external auditory meatus (EAML and EAMR, respectively) and the dorsum foramen magnum (DFM) were selected as suggested by previous research. The fourth point, ELSA, defined as the midpoint between the left and right foramen spinosum [32] was selected as the origin of the new Cartesian co-ordinate system. From the origin, 3D positional co-ordinates for the EAML, EAMR and DFM were determined [7].

**Fig. 5:**
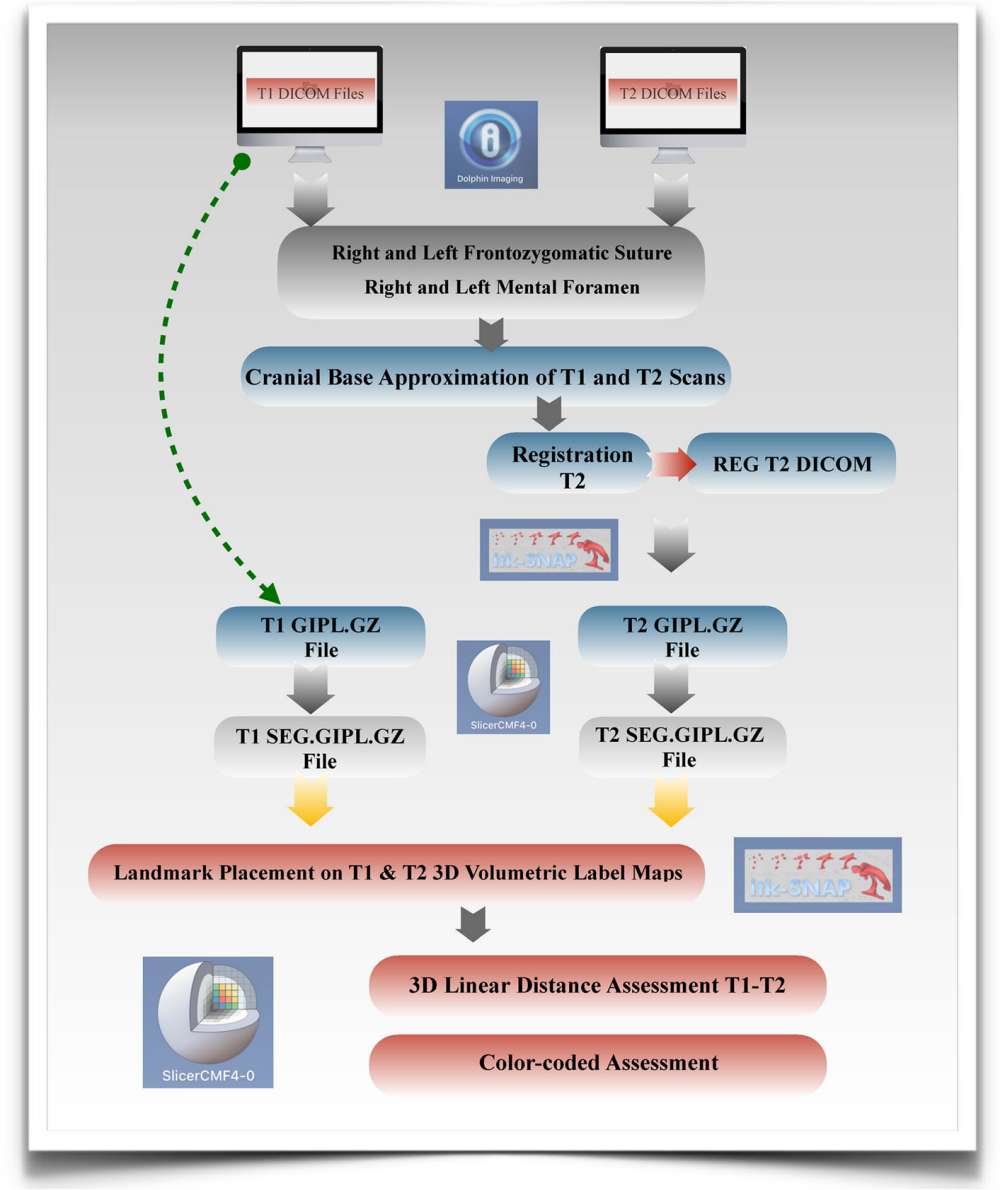
Flow Diagram Landmark-derived Method. Using AVIZO software, sagittal, axial and coronal multiplanar slices, as well as the 3D image reconstructions, were used to determine the position of the seven landmarks used to superimpose the T1 and T2 images; left and right auditory external meatus, left and right foramen spinosum, left and right foramen ovale and dorsum foramen magnum; as well as the ten landmarks use to assess reliability and measurement error. Once data was optimized in Matlab, linear distances between the 3D coordinates were calculated using the Euclidean distance formula in Excel

**Fig. 6:**
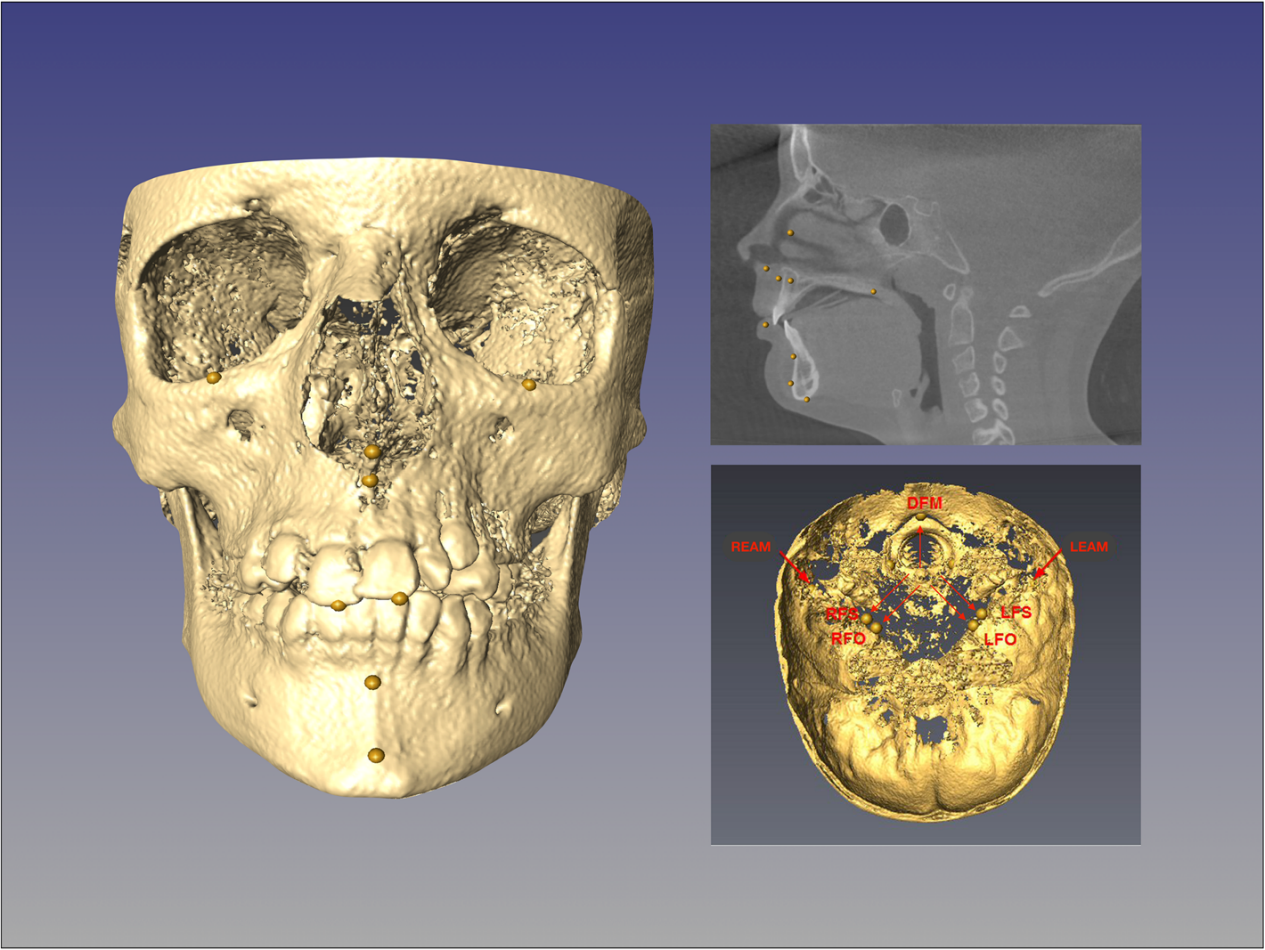
Landmark placement with landmark-derived method. Image reconstruction and sagittal slice with 0.5 mm yellow landmarks. Cranial base section with seven landmarks used for superimposing pre and post- treatment scans

The optimization formulation used in this study was the 6-point algorithm, that not only optimizes the location of the same three points (i.e. EAML, EAMR and DFM) as used in the 4-point algorithm but also includes both foramen ovale [right and left (FOR and FOL)] in each image [33, 34]. The addition of two extra landmarks (FOR and FOL) in the optimization analysis was shown to reduce the envelope of error when determining the co-ordinate system [7]. Once data was optimized, linear distances between the 3D coordinates were calculated using the Euclidean distance formula. Each landmark was included in multiple linear measurements of different orientations to be able to assess all dimensions (superior-inferior, anterior-posterior, right-left) (Figs. 5 and 6).

### Voxel-based – dolphin method

For each patient, T1 and T2 CBCT images were approximated using four landmarks located at the right and left frontozygomatic sutures and the right and left mental foramen and superimposed on the cranial base using voxel-based superimposition tool in Dolphin 3D (Chatsworth, CA -version 11.8.06.15 premium). The area of the cranial base used for superimposition was defined by a red box in the three different multiplanar views (axial, sagittal and coronal). The superimposition was achieved by moving the T2 image in relation to the T1 image creating a registered T2 image. No head orientation procedure was performed, as Dolphin software does not have the tool.

Then the slice views (axial, sagittal and coronal) were used to confirm the precision of Dolphin 3D superimposition. Once this step was completed, the registered post-treatment scans were exported as DICOM files and opened in ITK-Snap software to convert them into GIPL format similar to the procedure done with the CMFreg/Slicer method. 3D slicer was then used to segment the whole skull using Intensity Segmenter tool, with the same intensity level for all cases to remove any potential error due to the segmentation process. Thus, a surface model of post-treatment segmentation was created for each particular patient. Then T1 and T2 images were ready for landmarking using ITK-Snap.

**Fig. 7:**
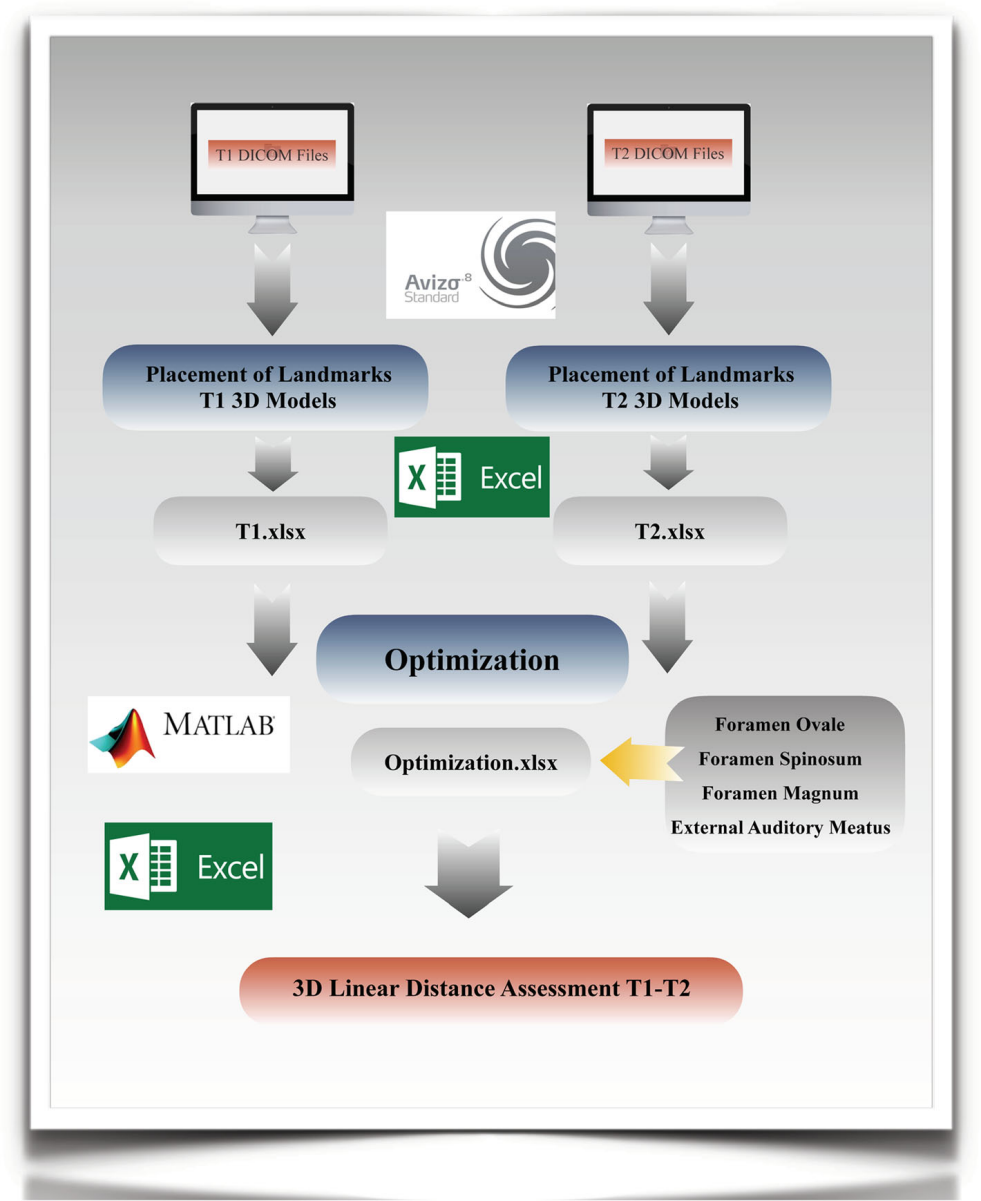
Flow Diagram Dolphin Method. T1 and T2 CBCT images are approximated using 4 landmarks located at the right and left frontozygomatic sutures and the right and left mental foramen and superimposed on the cranial base. Then the slice views (axial, sagittal and coronal) are used to confirm the precision of Dolphin 3D superimposition. Once this step is completed, the registered post-treatment scans are exported as DICOM files and opened in ITK-Snap software to convert them into GIPL format. After placing the defined landmarks to pre and post-treatment images, 3D surface models were created using 3D Slicer. 3D linear distances between T1 and T2 of corresponding landmarks are then quantified and color-coded maps are created

**Fig. 8:**
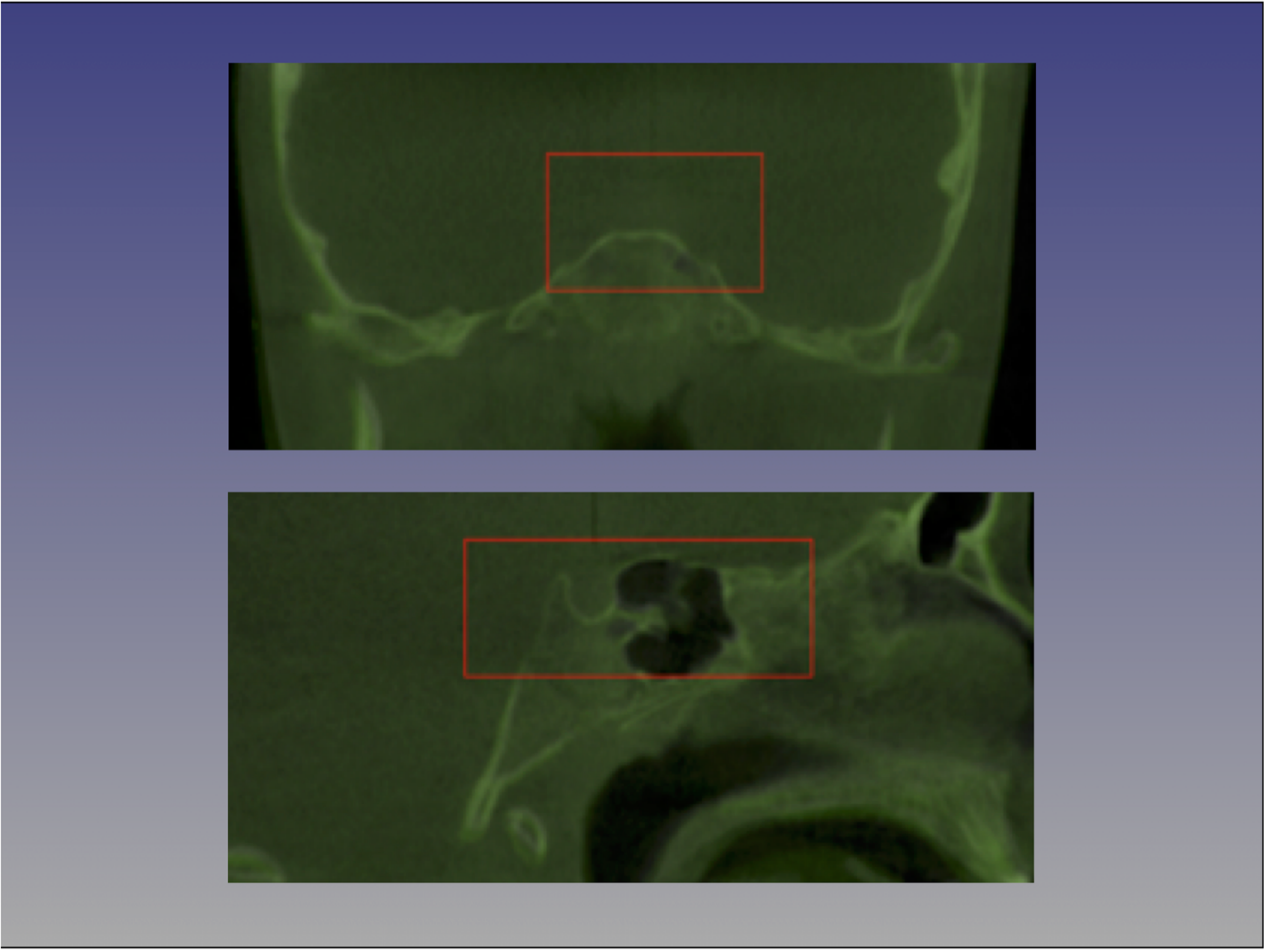
Cranial base approximation with Dolphin method. The area of the cranial base used for superimposition is defined by a red box in the three different multiplanar views (axial, sagittal and coronal), only coronal and sagittal slices are showing here. The superimposition is achieved by moving the T2 image in relation to the T1 image creating a registered T2 image

**Fig. 9:**
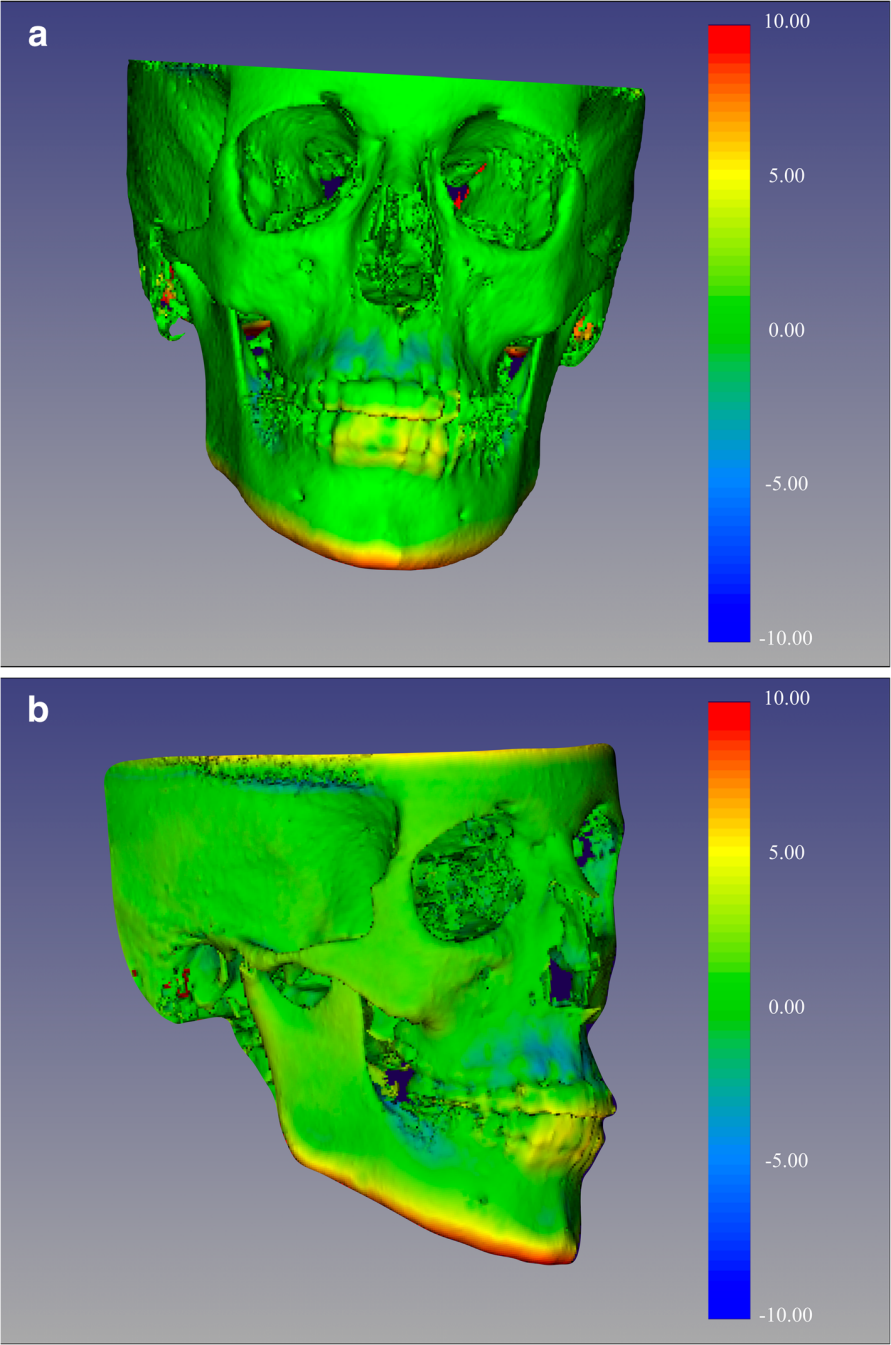
Color-coded maps with Dolphin method for visualization purposes only, not quantitative assessment. Frontal (Fig. 9a) and 45 (Fig. 9b) degrees views of the 3D color-coded maps showing the change in millimetres. As observed, no head orientation procedure has been performed, as Dolphin software does not have the tool

After placing the defined landmarks to pre and post-treatment images, 3D surface models were created using 3D Slicer for all the levels used in ITK-Snap. These models were utilized to measure the absolute differences between the pre and post-treatment images by applying the Q3DC function (Quantification of directional changes in each plane of the three planes of space). 3D linear distances between T1 and T2 of corresponding landmarks were quantified in the transversal (x-axis), antero-posterior (y-axis) and vertical (z-axis) direction (Figs. 7, 8 and 9).

#### Statistical analysis

For all tests, the statistical significance was set at *P*-value of 0.05.

### Intra-examiner reliability of 3D superimposition per method

Intraclass Correlation Coefficient (ICC) was used to measure the level of agreement between the two repeated measurements of 3D linear distances (difference between T2-T1) within each method by the principal investigator. Paired-sample T-test was performed to compare the means of corresponding measurements following the first and second superimpositions with registrations at the anterior cranial base and the first superimposition with registration at the cranial base and the landmark retracing only for both voxel-based methods (CMFreg/Slicer and Dolphin).

### Intra-examiner reliability of 3D superimposition among methods

ICC was used to assess the level of agreement between the measurements of 3D linear distances (difference between T2-T1) among all the three methods. 3D changes in the craniofacial complex with each method were assessed by one-way repeated measures analysis of variance (ANOVA) followed by post-hoc analysis.

## Results

A summary of results is presented in Tables 2, 3, 4, 5, 6, 7, 8, 9, 10 and 11.

**Table 2:** Intra-examiner reliability of linear measurements - voxel-based CMFreg/Slicer method

	Complete superimposition			Retracing landmarks only		
Distances	ICC	95% Confidence interval		ICC	95% Confidence interval	
		Lower bound	Upper bound		Lower bound	Upper bound
ANS T2-T1	0.933	0.731	0.983	0.940	0.757	0.985
A-Point T2-T1	0.866	0.462	0.967	0.894	0.573	0.974
PNS T2-T1	0.895	0.579	0.974	0.900	0.599	0.975
OrR T2-T1	0.834	0.331	0.959	0.899	0.593	0.975
OrL T2-T1	0.915	0.656	0.979	0.930	0.719	0.983
Menton T2-T1	0.990	0.959	0.997	0.998	0.990	0.999
B-Point T2-T1	0.968	0.869	0.992	0.973	0.893	0.993
GoR T2-T1	0.967	0.866	0.992	0.973	0.889	0.993
GoL T2-T1	0.904	0.613	0.976	0.942	0.765	0.985
Pg T2-T1	0.926	0.702	0.982	0.980	0.920	0.995

**Table 3:** Paired sample T-test - voxel-based CMFreg/Slicer method

	Complete superimposition				Retracing landmarks only			
Distances	Mean (mm)	95% Confidence interval		Sig.	Mean	95% Confidence interval		Sig.
		Lower	Upper			Lower	Upper	
ANS T2-T1	0.17	−0.321	0.661	0.454	− 0.03	− 0.456	0.392	0.867
A-Point T2-T1	0.33	− 0.407	1.073	0.336	0.47	−0.513	0.661	0.782
PNS T2-T1	0.34	−0.324	0.997	0.279	0.41	−0.133	0.956	0.122
OrR T2-T1	−0.21	− 0.579	0.154	0.222	−0.03	−0.351	0.288	0.828
OrL T2-T1	−0.01	− 0.424	0.233	0.526	−0.04	−0.334	0.251	0.755
Menton T2-T1	0.18	−0.165	0.528	0.266	−0.05	−0.226	0.126	0.534
B-Point T2-T1	−0.24	− 0.813	0.337	0.373	0.02	−0.510	0.558	0.921
GoR T2-T1	0.44	−0.319	0.921	0.064	0.24	−0.169	0.655	0.215
GoL T2-T1	0.67	−0.262	1.599	0.138	0.48	−0.251	1.219	0.17
Pg T2-T1	0.40	−0.441	1.246	0.308	0.20	−0.241	0.646	0.329

**Table 4:** Intra-examiner reliability of linear measurements - landmark-derived method

Distances	ICC	95% Confidence interval	
		Lower bound	Upper bound
ANS T2-T1	0.952	0.808	0.988
A-Point T2-T1	0.953	0.811	0.988
PNS T2-T1	0.712	−0.161	0.928
OrR T2-T1	0.913	0.650	0.978
OrL T2-T1	0.825	0.444	0.954
Menton T2-T1	0.955	0.831	0.989
B-Point T2-T1	0.926	0.732	0.981
GoR T2-T1	0.953	0.811	0.988
GoL T2-T1	0.948	0.805	0.987
Pg T2-T1	0.993	0.972	0.998

**Table 5:** Paired sample T-test - landmark-derived method

Distances	Mean (mm)	95% Confidence interval		Sig.
		Lower	Upper	
ANS T2-T1	0.74	−0.094	1.575	0.076
A-Point T2-T1	−0.09	− 0.985	0.804	0.824
PNS T2-T1	0.91	0.063	1.759	0.038
OrR T2-T1	0.71	−0.117	1.530	0.084
OrL T2-T1	0.89	0.061	1.710	0.038
Menton T2-T1	0.92	0.131	1.718	0.027
B-Point T2-T1	1.14	0.178	2.108	0.025
GoR T2-T1	0.74	− 0.160	1.634	0.096
GoL T2-T1	1.17	0.595	1.741	0.001
Pg T2-T1	−0.06	− 0.537	0.425	0.797

**Table 6:** Intra-examiner reliability of linear measurements - voxel-based dolphin method

	Complete superimposition			Landmarks only		
Distances	ICC	95% Confidence interval		ICC	95% Confidence interval	
		Lower bound	Upper bound		Lower bound	Upper bound
ANS T2-T1	0.959	0.835	0.99	0.968	0.870	0.992
A-Point T2-T1	0.911	0.641	0.978	0.916	0.661	0.979
PNS T2-T1	0.919	0.672	0.98	0.920	0.678	0.980
OrR T2-T1	0.905	0.616	0.976	0.936	0.741	0.984
OrL T2-T1	0.903	0.611	0.976	0.920	0.679	0.980
Menton T2-T1	0.993	0.970	0.998	0.995	0.978	0.999
B-Point T2-T1	0.949	0.793	0.987	0.959	0.834	0.990
GoR T2-T1	0.971	0.883	0.993	0.974	0.896	0.994
GoL T2-T1	0.943	0.772	0.986	0.956	0.823	0.989
Pg T2-T1	0.979	0.917	0.995	0.988	0.952	0.997

**Table 7:** Paired sample T-test - voxel-based dolphin method

	Complete superimposition				Landmarks only			
Distances	Mean (mm)	95% Confidence interval		Sig.	Mean	95% Confidence interval		Sig.
		Lower	Upper			Lower	Upper	
ANS T2-T1	0.13	−0.275	0.539	0.483	0.09	−0.264	0.441	0.582
A-Point T2-T1	0.19	− 0.375	0.753	0.468	−0.21	− 0.690	0.262	0.335
PNS T2-T1	−0.02	−0.294	0.254	0.871	−0.03	−0.370	0.302	0.823
OrR T2-T1	0.09	−0.175	0.360	0.454	−0.01	−0.249	0.237	0.955
OrL T2-T1	−0.03	−0.312	0.246	0.795	−0.02	−0.285	0.235	0.834
Menton T2-T1	−0.04	−0.350	0.272	0.781	−0.16	−0.431	0.119	0.232
B-Point T2-T1	0.29	−0.393	0.970	0.364	0.09	−0.508	0.691	0.738
GoR T2-T1	−0.04	−0.448	0.374	0.842	0.26	−0.152	0.663	0.190
GoL T2-T1	−0.03	−0.723	0.654	0.913	−0.03	−0.634	0.564	0.898
Pg T2-T1	0.40	−0.062	0.863	0.082	0.11	−0.251	0.465	0.517

**Table 8:** Intra-examiner reliability of linear measurements - three superimposition methods

	Three superimposition methods			Voxel-based (CMFreg/Slicer and dolphin)		
Distances	ICC	95% Confidence interval		ICC	95% Confidence interval	
		Lower bound	Upper bound		Lower bound	Upper bound
ANS T2-T1	0.307	−0.059	0.589	0.885	0.777	0.941
A-Point T2-T1	0.389	−0.023	0.662	0.863	0.733	0.93
PNS T2-T1	0.480	0.136	0.709	0.545	0.101	0.769
OrR T2-T1	−0.071	−0.337	0.23	0.596	0.211	0.793
OrL T2-T1	0.267	−0.074	0.551	0.741	0.492	0.868
Menton T2-T1	0.659	0.197	0.845	0.904	0.901	0.974
B-Point T2-T1	0.549	0.139	0.769	0.943	0.889	0.971
GoR T2-T1	0.646	0.374	0.809	0.972	0.945	0.986
GoL T2-T1	0.759	0.574	0.87	0.787	0.582	0.892
Pg T2-T1	0.402	0.029	0.659	0.919	0.841	0.959

**Table 9:** Intra-examiner reliability of linear measurements - three superimposition methods

	Voxel-based CMFreg/Slicer and landmark-derived			Voxel-based dolphin and landmark-derived		
Distances	ICC	95% Confidence interval		ICC	95% Confidence interval	
		Lower bound	Upper bound		Lower bound	Upper bound
ANS T2-T1	0.119	−0.201	0.422	0.143	− 0.182	0.446
A-Point T2-T1	0.231	− 0.194	0.555	0.210	−0.195	0.529
PNS T2-T1	0.289	−0.231	0.611	0.353	−0.158	0.656
OrR T2-T1	−0.137	−0.539	0.254	−0.081	− 0.377	0.241
OrL T2-T1	0.167	−0.177	0.475	0.100	−0.154	0.372
Menton T2-T1	0.460	−0.176	0.751	0.480	−0.201	0.772
B-Point T2-T1	0.348	−0.171	0.656	0.335	−0.178	0.646
GoR T2-T1	0.394	−0.094	0.676	0.406	−0.077	0.684
GoL T2-T1	0.538	0.125	0.76	0.717	0.313	0.871
Pg T2-T1	0.252	−0.214	0.574	0.257	−0.209	0.577

**Table 10:** One-way repeated measures anova - pairwise comparisons

Landmarks	Superimposition method	95% Confidence interval			
		Mean (mm)	Lower bound	Upper bound	p-value
ANS	Voxel-Based CMFreg/Slicer Method - Landmark-derived Method	3.312	2.119	4.505	0.000
	Voxel-Based CMFreg/Slicer Method - Voxel-based Dolphin Method	0.148	−0.211	0.508	0.920
	Voxel-Based Dolphin Method - Landmark-derived Method	3.460	2.327	4.594	0.000
A-Point	Voxel-Based CMFreg/Slicer Method - Landmark-derived Method	3.306	2.201	4.412	0.000
	Voxel-Based CMFreg/Slicer Method - Voxel-based Dolphin Method	0.129	−0.256	0.515	1.000
	Voxel-Based Dolphin Method - Landmark-derived Method	3.177	2.038	4.316	0.000
PNS	Voxel-Based CMFreg/Slicer Method - Landmark-derived Method	1.021	0.239	1.804	0.007
	Voxel-Based CMFreg/Slicer Method - Voxel-based Dolphin Method	0.116	−0.474	0.707	1.000
	Voxel-Based Dolphin Method - Landmark-derived Method	1.138	0.594	1.681	0.000
Orbitale Right	Voxel-Based CMFreg/Slicer Method - Landmark-derived Method	2.731	1.622	3.840	0.000
	Voxel-Based CMFreg/Slicer Method - Voxel-based Dolphin Method	0.163	−0.257	0.583	1.000
	Voxel-Based Dolphin Method - Landmark-derived Method	2.894	1.876	3.912	0.000
Orbitale Left	Voxel-Based CMFreg/Slicer Method - Landmark-derived Method	2.927	2.026	3.829	0.000
	Voxel-Based CMFreg/Slicer Method - Voxel-based Dolphin Method	0.089	−0.308	0.486	1.000
	Voxel-Based Dolphin Method - Landmark-derived Method	3.016	2.164	3.868	0.000
Menton	Voxel-Based CMFreg/Slicer Method - Landmark-derived Method	3.521	2.155	4.886	0.000
	Voxel-Based CMFreg/Slicer Method - Voxel-based Dolphin Method	0.144	−0.286	0.573	1.000
	Voxel-Based Dolphin Method - Landmark-derived Method	3.664	2.372	4.957	0.000
B-Point	Voxel-Based CMFreg/Slicer Method - Landmark-derived Method	3.371	1.880	4.862	0.000
	Voxel-Based CMFreg/Slicer Method - Voxel-based Dolphin Method	0.094	−0.305	0.494	1.000
	Voxel-Based Dolphin Method - Landmark-derived Method	3.466	1.965	4.966	0.000
Gonion Right	Voxel-Based CMFreg/Slicer Method - Landmark-derived Method	1.517	0.416	2.618	0.004
	Voxel-Based CMFreg/Slicer Method - Voxel-based Dolphin Method	0.068	−0.160	0.297	1.000
	Voxel-Based Dolphin Method - Landmark-derived Method	1.449	0.345	2.552	0.007
Gonion Left	Voxel-Based CMFreg/Slicer Method - Landmark-derived Method	0.993	−0.008	1.995	0.052
	Voxel-Based CMFreg/Slicer Method - Voxel-based Dolphin Method	0.127	−0.578	0.832	1.000
	Voxel-Based Dolphin Method - Landmark-derived Method	1.120	0.415	1.825	0.001
Pogonion	Voxel-Based CMFreg/Slicer Method - Landmark-derived Method	5.093	2.432	7.754	0.000
	Voxel-Based CMFreg/Slicer Method - Voxel-based Dolphin Method	0.010	−0.467	0.487	1.000
	Voxel-Based Dolphin Method - Landmark-derived Method	5.103	2.486	7.719	0.000

**Table 11:** Advantages and disadvantages of 3d superimposition methods

Superimposition methods			
Features	CMFreg/Slicer	Dolphin	Landmark-derived
Processing Time	Up to 3 h from segmentation to color-coded maps. Cranial base registration itself takes from 5 to 60 min	Up to 50 min if numerical assessment and color–coded maps for visual evaluation are required. Cranial base registration itself takes about10 minutes, but it often fails, requiring repetition, which increasing time	Up to 35 min from landmarking to numerical assessment. Approximately 15 min for landmarking only. Numerical assessment requires another 15–20 min
Complexity	Highly complex for a naïve user. Multiple steps and two different software programs are used throughout the process	Easy to use but utilizes a volume cube for reference not only stable structures. 3D surfaces visual and quantitative evaluations after registration steps require similar steps to CMFreg/Slicer and need to be followed using Slicer and ITK-Snap software programs	Easy to use. Only landmark placement is required
Convenience/Access	Open-source software programs. 3D Slicer and ITK-Snap	License required	License required
Visual Assessment	Superimposition of surface models and color-coded maps for more thorough visual evaluation	Superimposition of surface models and color-coded maps using other software for more thorough visual evaluation	Not available
Reliability	Excellent reliability	Excellent reliability	Good reliability
Measurement Error	Less than 0.7 mm	Less than 0.4 mm	Less than 1.2 mm when assessed reliability. However, there was a magnified error when compared to the voxel-based methods
Accuracy	No gold standard available	No gold standard available	No gold standard available

### Intra-examiner reliability of 3D superimposition per method

#### Voxel-based CMFreg/slicer method: first and second Cranial Base superimposition

Using ten pre-determined 3D linear distances, good to excellent agreement for intra-examiner reliability was found on all skeletal landmarks as indicated by an ICC ≥ 0.904. All these ICC values were considered acceptable; however, lower bound of CI of two landmarks (APoint and OrR) were below 0.50 (Table 2).

#### Voxel-based CMFreg/slicer method: first Cranial Base superimposition and landmark retracing only

Good to excellent agreement for intra-examiner reliability was found on all skeletal landmarks in the 3D measurements as indicated by an ICC ≥ 0.900. All lower bound of CI were above 0.50 (Table 2).

Table 3 shows the differences between the first and second superimposition with registration at the anterior cranial base. Mean differences between both superimpositions were less than 0.67 mm. No statistically significant differences were found at any landmark (*P*-values > 0.05).

Table 3 also shows the differences between the first superimposition with registration at the anterior cranial base and the landmark retracing. Mean differences between both trials were less than 0.74 mm. No statistically significant differences were found at any landmark (*P*-values > 0.05).

#### Landmark-derived method

Excellent agreement for intra-examiner reliability was found on eight skeletal landmarks in the 3D measurements as indicated by an ICC ≥ 0.913. OrL and PNS showed good and moderate intra-examiner reliability respectively, ICC ≥ 0.712. All these ICC values are considered acceptable; however, lower bound of CI of two landmarks (OrL and PNS) were below 0.50 (Table 4).

Mean differences between the first and second superimpositions were as high as 1.168 mm. Statistically significant differences were found at five skeletal landmarks: PNS, OrL, Menton, BPoint, and GoL (*P*-values < 0.05) (Table 5).

#### Voxel-based dolphin method: first and second Cranial Base superimposition

Excellent agreement for intra-examiner reliability was found on all skeletal landmarks in the 3D measurements as indicated by an ICC ≥ 0.905 (Table 6).

#### Voxel-based dolphin method: first Cranial Base superimposition and landmark retracing only

Excellent agreement for the intra-examiner reliability was observed on all skeletal landmarks in the 3D measurements as indicated by an ICC ≥ 0.916, when only landmarks were retraced (Table 6).

Table 7 shows the differences between the first and second superimposition with registration at the anterior cranial base. Mean differences between both superimpositions were less than 0.4 mm. No statistically significant differences were found at any skeletal landmark (*P*-values > 0.05).

Table 7 also shows the differences between the first superimposition with registration at the anterior cranial base and the landmark retracing. Mean differences between both trials were less than 0.26 mm. No statistically significant differences were found at any skeletal landmark (*P*-values > 0.05).

### Intra-examiner reliability of 3D superimposition among methods

Good agreement for the intra-examiner reliability was observed only at GoL, ICC = 0.759 when the three 3D superimposition methods were evaluated. Menton, BPoint and GoR showed moderate agreement as indicated by an ICC ≥ 0.549 (Table 8).

When assessing both voxel-based methods (CMFreg/Slicer and Dolphin), excellent agreement for intra-examiner reliability was noted on four skeletal landmarks (Me, BPoint, GoR and Pg) in the 3D measurements as indicated by an ICC ≥ 0.904 (Table 8). However, when assessing the voxel-based CMFreg/Slicer and the Landmark-derived methods, moderate agreement was found only at GoL, ICC = 0.538. The rest of skeletal landmarks showed poor agreement as indicated by an ICC ≥ − 0.137 (Table 9). A similar trend was observed when assessing the voxel-based Dolphin and the Landmark-derived methods, moderate agreement for the intra-examiner reliability only at GoL, ICC = 0.717. The rest of the skeletal landmarks showed poor agreement as indicated by an ICC ≥ − 0.081 (Table 9).

The one-way repeated measurements ANOVA revealed evidence of a statistically significant difference between the mean of distances T2-T1 when comparing CMFreg/Slicer method to Landmark-derived method and when comparing the Dolphin method to the Landmark-derived method in the overall 3D at all dependent variables (Table 10).

## Discussion

Historically, cranial base superimposition of serial lateral cephalograms has provided clinicians with a visual assessment of overall hard and soft tissue changes resulting from treatment, either orthodontic, orthopedic or orthognathic surgery; and/or growth during a time frame. One of the major disadvantages of using a conventional cephalometric analysis is that 3D information is depicted as 2D data and often limited to midline structures. Improvements in image registration algorithms have led to the development of new methods for CBCT volume superimposition to overcome the issues faced with generated 2D images.

The challenge of image registration is to superimpose CBCT volumes of patients with craniofacial changes due to the normal growth and/or treatment response at different time-points. In these situations, the different CBCT volumes may have dissimilar imaging acquisition, field of view, and dental/skeletal components modified by growth and/or treatments, making the registration process more difficult and prone to failure. Therefore, this study aimed to compare three commonly used 3D superimposition methods and determine if they can reliably be used to superimpose T1 and T2 CBCT images of growing patients registered at the anterior cranial base and if there is any difference among them.

The reliability of the three 3D superimposition methods was tested in this study by calculating the mean linear distances between the two models (T2-T1) at ten different anatomic regions. When the methods were analyzed individually, the ICC results showed good to excellent agreement for the intra-examiner reliability with CMFreg/Slicer and landmark-derived methods, and excellent intra-examiner reliability when CBCT images were superimposed with Dolphin method. The slightly higher agreement observed with the Dolphin method could just be a reflection of the examiner’s expertise since this was the last method assessed. Similar although less powerful results were reported by Nada et al. [35], who tested the reproducibility of CBCT superimposition on the anterior cranial base and the zygomatic arches using voxel-based image registration of 3D CBCT scans from sixteen adult patients who underwent combined surgical orthodontic treatment. When the models were registered at the anterior cranial base, intra-observer reliability was reported to be moderate to good between the repeated superimpositions: the ICC ranged between 0.53 and 0.94 and the mean distances between the two models registered on the zygomatic arch remained within 0.5 mm. Likewise, Cevidanes et al. [22] studied the variability between observers in quantification of treatment outcome only using color-coded distance maps for different anatomic regions on 3D CBCT models registered on the anterior cranial base using a voxel-method method. They reported an inter-examiner range of measurements across anatomic regions equal or less than 0.5 mm, which they considered to be clinically insignificant.

The reproducibility of the registration was also tested on both voxel-based (CMFreg/Slicer and Dolphin) methods. There were no evident differences found between the first and second cranial base registrations and the retracing landmarks only, as demonstrated by an excellent agreement for the intra-examiner reliability. In addition, paired t-tests showed no statistical significance with mean differences between both the superimposition and retracing landmarks only. Since differences ≤0.4 mm are not likely clinically significant, the registration process of CMFreg/Slicer and Dolphin methods can be considered clinically reproducible. These results are in agreement with the reports from Cevidanes et al., [22] who assessed cranial base superimposition in growing patients and Nguyen et al. [36] and Ruellas et al. [30] who tested regional superimpositions demonstrating a similar range in their findings.

On the other hand, when assessing reliability among the three methods, the ICC demonstrated less powerful agreement with a wide range of confidence interval. ICC values were the lowest when comparing the landmark-derived method and the voxel-based (CMFreg/Slicer and Dolphin) methods. Moderate to excellent agreement; however, was observed for the intra-examiner reliability when comparing the voxel-based methods against each other; even though the head orientation procedure was not performed with the Dolphin method. Ruellas et al. [31] have shown that the amount of directional change in each plane of 3D space is strongly influenced by head orientation, and the precise assessment of direction of change requires a common 3D coordinate system.

From the results of this study, the three 3D superimposition methods demonstrated an overall 3D change in the craniofacial complex during an average of 24 months of evaluation (mean age of 12.4 years - CVM 3–4 at initial records). Both voxel-based methods (CMFreg/Slicer and Dolphin) showed similar mean differences between T1 and T2 images with no statistical significance in their differences. On the other hand, the landmark-derived method exhibited mean differences as high as twice as the mean differences obtained with any of the voxel-based methods in the overall 3D assessment. When the methods assessed the changes at each landmark per components, eight skeletal landmarks (ANS, APoint, PNS, Menton, Bpoint, GoR, GoL and Pg) showed the highest variation in the superior-inferior component, with inferior direction, and two skeletal landmarks (OrR and OrL) in the antero-posterior component, with anterior drift. Similar to the overall 3D evaluation, the landmark-derived method exhibited the highest mean differences when assessed per component, being the superior-inferior component that demonstrated the most substantial variation (Appendices I – II).

According to the present study, the landmark-derived method generated magnified errors since the 3D linear distances were higher when compared to the other two methods in all the defined landmarks. Although the method showed moderate to excellent agreement for the intra-examiner reliability when assessed individually, poor to moderate agreement was observed when all the methods were evaluated simultaneously. These results contradict the findings from DeCesare [7] study, who reported a reduced envelope of error using the 6-point correction algorithm optimized analysis instead of the 4-point when determining the co-ordinate system. Although, the landmark-derived registration method uses a number of landmarks as reference and they could be susceptible to landmark identification errors, reliability in landmark identification was determined to be adequate. Therefore, a potential reason for the reduced reliability and increased measurement error may be the lack of stability of the reference areas, as the landmarks used to superimpose the pre- and post-treatment images are located in the medial and posterior cranial base, which are known as unstable areas due to growth and remodeling that occurs during childhood and adolescence [1, 14, 37, 38].

The magnitude of variation obtained with both voxel-based methods (CMFreg/Slicer and Dolphin) appears to be within the range of change observed by previous research [39–45]. However, as none of these methods are considered the gold standard for 3D superimposition – the realistic validity standard to be compared to; the accuracy of the results cannot be determined. Therefore, it is unknown if the amount of change generated by the two voxel-based (CMFreg/Slicer and Dolphin) methods is closer to the real value or it is the landmark method the one that is closer to the truth. Nevertheless, it is a good start to know that two similar computing-based superimposition methods generated quite similar measurements (Table 11). In addition, as the included individuals had orthodontic treatment, it is not possible to verify if the amount of change seen at the specific landmarks in the maxilla and mandible was due to growth only, or it was a combination of growth and treatment effects. Consequently, even with the availability of 3D imaging, quantification of growth/treatment is still an area for research.

### Limitations

The biggest limitation of this study is the lack of a gold standard (ground truth) for 3D superimposition. Thus, although two out of the three methods tested in this study showed very minor differences between them and the mean differences were not statistically significant, it is not possible to determine the accuracy of the results.

Another important limitation is the use of a single investigator and the significant learning curve that all of the three 3D superimposition methods used in this study required. CMFreg/Slicer method had the highest level of complexity among all the three methods and used two different software programs (3D Slicer and ITK-Snap) throughout the process. Although it includes systematic steps to obtain a high level of precision, it is highly time-consuming. Dolphin method, on the other hand, is faster and user-friendlier, however, to quantify changes, scans are required to be loaded in ITKSnap for landmark placement and then measure using Q3DC tool in 3D Slicer. These additional steps increase the working time and process complexity. The landmark-derived method appears to be simpler, since it only requires landmark placement similar as in a 2D cephalometric analysis, although in a 3D image. However, the software requires some expertise and it does not allow viewing the landmarks in all three planes at the same time, so the researcher requires to change planes continuously to check landmark position in all the different planes.

The possible effect of the segmentation process, the different software programs used for the superimposition as well as the landmark identification are sources of measurement error in 3D radiographic imaging.

The surface model construction in CBCT is based on the voxel-based data. A threshold value specifies each structure whether it is bone or soft tissue. The threshold value and gray value entered by the operator in to the CBCT machine determines the image accuracy. Also, the CBCT imaging lacks beam homogeneity which means that the gray value of the voxels of the CBCT of the same individual at different time points differ [46, 47].

The potential impact due to limited resolution of the CBCT data (0.3 mm) on the overall precision is not possible to quantify in this study as all three methods used the same data set. However, increasing imaging resolution and maintaining size of the scan would increase the radiation dose.

Finally, due to the lack of a control group differentiation between the treatment and normal growth changes was not possible.

## Conclusions

Findings of the research indicate good to excellent intra-examiner reliability of the three 3D superimposition methods when assessed individually. However, when assessing reliability among the three methods, the ICC demonstrated less powerful agreement with a wide range of confidence interval. ICC values were the lowest when comparing the landmark-based method and the voxel-based (CMFreg/Slicer and Dolphin) methods. Moderate to excellent agreement was observed for the intra-examiner reliability when comparing the voxel-based methods against each other. Two of the three methods (CMFreg/Slicer and Dolphin) used in this study showed similar mean differences; however, the accuracy of the results could not be determined since none of them have been considered the gold standard for 3D superimposition in growing patients. The landmark-based method generated the highest measurement error among the three methods.

## Data Availability

The datasets used and/or analysed during the current study are available from the corresponding author on reasonable request.

## Appendix 1

**Table 12:** Descriptives of repeated measures for all distances

Landmarks	Superimposition method	Mean (mm)	95% Confidence interval	
			Lower bound	Upper bound
ANS	Voxel-based CMFreg/Slicer Method	2.688	2.207	3.170
	Landmark-derived Method	6.000	5.083	6.917
	Voxel-based Dolphin Method	2.540	2.117	2.963
A-Point	Voxel-based CMFreg/Slicer Method	2.541	2.098	2.985
	Landmark-derived Method	5.848	4.896	6.799
	Voxel-based Dolphin Method	2.671	2.220	3.122
PNS	Voxel-based CMFreg/Slicer Method	1.850	1.312	2.388
	Landmark-derived Method	2.871	2.410	3.333
	Voxel-based Dolphin Method	1.734	1.467	2.001
OrR	Voxel-based CMFreg/Slicer Method	1.583	1.204	1.963
	Landmark-derived Method	4.314	3.566	5.062
	Voxel-based Dolphin Method	1.420	1.184	1.656
OrL	Voxel-based CMFreg/Slicer Method	1.543	1.113	1.974
	Landmark-derived Method	4.471	3.768	5.173
	Voxel-based Dolphin Method	1.454	1.205	1.704
Me	Voxel-based CMFreg/Slicer Method	5.690	4.892	6.488
	Landmark-derived Method	9.210	7.912	10.509
	Voxel-based Dolphin Method	5.546	4.771	6.321
B-Point	Voxel-based CMFreg/Slicer Method	4.595	3.904	5.285
	Landmark-derived Method	7.966	6.663	9.268
	Voxel-based Dolphin Method	4.500	3.810	5.191
GoR	Voxel-based CMFreg/Slicer Method	4.623	4.075	5.172
	Landmark-derived Method	6.141	5.233	7.048
	Voxel-based Dolphin Method	4.692	4.129	5.255
GoL	Voxel-based CMFreg/Slicer Method	4.576	3.836	5.315
	Landmark-derived Method	5.569	4.833	6.305
	Voxel-based Dolphin Method	4.449	3.845	5.052
Pg	Voxel-based CMFreg/Slicer Method	5.086	4.340	5.832
	Landmark-derived Method	10.179	7.873	12.495
	Voxel-based Dolphin Method	5.076	4.434	5.718

## Appendix 2

**Table 13:** Descriptive statistics - three methods per components

			95% Confidence interval	
Landmarks	Superimposition method	Mean (mm)	Lower bound	Upper bound
ANS R-L	Voxel-Based CMFreg/Slicer Method	0.057	−0.140	0.254
	Landmark-derived Method	0.185	−0.622	0.992
	Voxel-based Dolphin Method	0.096	−0.146	0.338
ANS A-P	Voxel-Based CMFreg/Slicer Method	1.103	0.582	1.625
	Landmark-derived Method	1.146	0.435	1.856
	Voxel-based Dolphin Method	1.210	0.730	1.690
ANS S-I	Voxel-Based CMFreg/Slicer Method	−1.717	−2.130	−1.305
	Landmark-derived Method	−4.373	−5.604	−3.142
	Voxel-based Dolphin Method	−1.680	−2.060	− 1.301
A-Point R-L	Voxel-Based CMFreg/Slicer Method	0.036	−0.165	0.237
	Landmark-derived Method	0.223	−0.546	0.992
	Voxel-based Dolphin Method	0.127	−0.079	0.333
A-Point A-P	Voxel-Based CMFreg/Slicer Method	0.628	0.279	0.978
	Landmark-derived Method	0.534	−0.081	1.150
	Voxel-based Dolphin Method	0.786	0.430	1.141
A-Point S-I	Voxel-Based CMFreg/Slicer Method	−1.900	−2.410	−1.390
	Landmark-derived Method	−4.503	−5.742	−3.265
	Voxel-based Dolphin Method	−2.076	−2.617	−1.536
PNS R-L	Voxel-Based CMFreg/Slicer Method	−0.064	−0.238	0.110
	Landmark-derived Method	−0.079	−0.398	0.240
	Voxel-based Dolphin Method	0.092	−0.108	0.292
PNS A-P	Voxel-Based CMFreg/Slicer Method	−0.151	− 0.515	0.214
	Landmark-derived Method	−0.650	−1.156	−0.144
	Voxel-based Dolphin Method	−0.124	− 0.522	0.274
PNS S-I	Voxel-Based CMFreg/Slicer Method	−1.217	− 1.528	−0.905
	Landmark-derived Method	−1.974	−2.537	−1.411
	Voxel-based Dolphin Method	−1.115	−1.394	−0.836
Orbitale Right R-L	Voxel-Based CMFreg/Slicer Method	0.192	−0.045	0.428
	Landmark-derived Method	0.353	−0.455	1.162
	Voxel-based Dolphin Method	0.018	−0.083	0.120
Orbitale Right A-P	Voxel-Based CMFreg/Slicer Method	0.964	0.667	1.262
	Landmark-derived Method	1.164	0.644	1.685
	Voxel-based Dolphin Method	1.076	0.788	1.363
Orbitale Right S-I	Voxel-Based CMFreg/Slicer Method	0.126	0.108	0.360
	Landmark-derived Method	2.054	0.980	3.129
	Voxel-based Dolphin Method	0.105	0.145	0.354
Orbitale Left R-L	Voxel-Based CMFreg/Slicer Method	−0.074	−0.213	0.064
	Landmark-derived Method	−0.404	−1.268	0.460
	Voxel-based Dolphin Method	−0.084	− 0.217	0.049
Orbitale Left A-P	Voxel-Based CMFreg/Slicer Method	1.055	0.735	1.376
	Landmark-derived Method	1.358	0.938	1.778
	Voxel-based Dolphin Method	1.217	0.942	1.492
Orbitale Left S-I	Voxel-Based CMFreg/Slicer Method	0.198	0.017	0.413
	Landmark-derived Method	2.057	0.967	3.148
	Voxel-based Dolphin Method	0.242	0.053	0.431
Menton R-L	Voxel-Based CMFreg/Slicer Method	−0.117	−0.445	0.211
	Landmark-derived Method	0.247	−0.691	1.185
	Voxel-based Dolphin Method	0.013	−0.323	0.349
Menton A-P	Voxel-Based CMFreg/Slicer Method	0.900	0.159	1.641
	Landmark-derived Method	1.122	0.521	2.765
	Voxel-based Dolphin Method	0.994	0.272	1.716
Menton S-I	Voxel-Based CMFreg/Slicer Method	−4.922	−5.680	−4.163
	Landmark-derived Method	−7.144	−8.534	−5.755
	Voxel-based Dolphin Method	−4.964	−5.719	− 4.208
B-Point R-L	Voxel-Based CMFreg/Slicer Method	−0.042	−0.402	0.318
	Landmark-derived Method	0.132	−0.832	1.097
	Voxel-based Dolphin Method	0.000	−0.353	0.353
B-Point A-P	Voxel-Based CMFreg/Slicer Method	0.711	0.064	1.358
	Landmark-derived Method	0.794	0.559	2.148
	Voxel-based Dolphin Method	0.786	0.144	1.428
B-Point S-I	Voxel-Based CMFreg/Slicer Method	−3.812	−4.535	−3.090
	Landmark-derived Method	−5.854	−7.369	−4.339
	Voxel-based Dolphin Method	−3.832	−4.555	− 3.109
Gonion Right R-L	Voxel-Based CMFreg/Slicer Method	1.296	0.996	1.596
	Landmark-derived Method	1.496	1.078	1.915
	Voxel-based Dolphin Method	1.404	1.109	1.699
Gonion Right A-P	Voxel-Based CMFreg/Slicer Method	−1.205	−1.764	−0.646
	Landmark-derived Method	−2.651	−3.994	−1.308
	Voxel-based Dolphin Method	−0.977	−1.504	−0.451
Gonion Right S-I	Voxel-Based CMFreg/Slicer Method	−3.536	−4.252	−2.820
	Landmark-derived Method	−3.638	−4.435	−2.840
	Voxel-based Dolphin Method	−3.841	−4.522	−3.160
Gonion Left R-L	Voxel-Based CMFreg/Slicer Method	−1.230	−1.519	−0.941
	Landmark-derived Method	−1.130	−1.462	−0.797
	Voxel-based Dolphin Method	−1.069	−1.399	−0.740
Gonion Left A-P	Voxel-Based CMFreg/Slicer Method	−1.396	−2.042	−0.750
	Landmark-derived Method	−2.135	−3.083	−1.186
	Voxel-based Dolphin Method	−0.989	−1.522	−0.455
Gonion Left S-I	Voxel-Based CMFreg/Slicer Method	−3.138	−3.857	−2.419
	Landmark-derived Method	−3.929	−4.753	−3.105
	Voxel-based Dolphin Method	−3.696	−4.362	−3.031
Pogonion R-L	Voxel-Based CMFreg/Slicer Method	−0.037	−0.381	0.306
	Landmark-derived Method	0.063	−1.068	1.193
	Voxel-based Dolphin Method	−0.001	−0.329	0.327
Pogonion A-P	Voxel-Based CMFreg/Slicer Method	1.025	0.322	1.729
	Landmark-derived Method	0.115	−1.753	1.983
	Voxel-based Dolphin Method	1.095	0.422	1.769
Pogonion S-I	Voxel-Based CMFreg/Slicer Method	−4.216	−4.949	−3.483
	Landmark-derived Method	−5.746	−8.723	−2.770
	Voxel-based Dolphin Method	−4.425	−5.087	−3.762

## References

[1] American Board of Orthodontics 2D Cranial Base Superimposition. https://www.americanboardortho.com. Accessed on March 10, 2018.

[2] Jacobson A, Jacobson R. Radiographic Cephalometry. Second Edition ed: Quintessence; 2006.

[3] Duterloo H, Planché P. Handbook of cephalometric superimposition Hanover Park, IL: Quintessence Pub., c2011.; 2011.

[4] Arat ZM, Türkkahraman H, English JD, Gallerano RL, Boley JC (2010). Longitudinal growth changes of the cranial base from puberty to adulthood. A comparison of different superimposition methods. Angle Orthod.

[5] De Clerck H, Nguyen T, de Paula LK, Cevidanes L (2012). Three-dimensional assessment of mandibular and glenoid fossa changes after bone-anchored class III intermaxillary traction. Am J Orthod Dentofac Orthop.

[6] Gu Y, McNamara JA (2008). Cephalometric superimpositions. A comparison of anatomical and metallic implant methods. Angle Orthod.

[7] DeCesare A, Secanell M, Lagravère M, Carey J (2013). Multiobjective optimization framework for landmark measurement error correction in three-dimensional cephalometric tomography. Dentomaxillofac Radiol.

[8] Björk A (1968). The use of metallic implants in the study of facial growth in children: method and application. Am J Phys Anthropol.

[9] Melsen B (1974). The cranial base. Acta Odontol Scand.

[10] Melsen B, Melsen F (1982). The postnatal development of the palatomaxillary region studied on human autopsy material. Am J Orthod.

[11] Björk A, Skieller V (1983). Normal and abnormal growth of the mandible. A synthesis of longitudinal cephalometric implant studies over a period of 25 years. Eur J Orthod.

[12] Cevidanes LH, Motta A, Proffit WR, Ackerman JL, Styner M (2010). Cranial base superimposition for 3-dimensional evaluation of soft-tissue changes. Am J Orthod Dentofac Orthop.

[13] Ghafari J, Engel FE, Laster LL (1987). Cephalometric superimposition on the cranial base: a review and a comparison of four methods. Am J Orthod Dentofac Orthop.

[14] Afrand M (2015). Anterior and middle cranial base growth and development changes as assessed through CBCT imaging in adolescents [dissertation].

[15] Berkowitz S (1999). A multicenter retrospective 3D study of serial complete unilateral cleft lip and palate and complete bilateral cleft lip and palate casts to evaluate treatment: part 1—the participating institutions and research aims. Cleft Palate Craniofac J.

[16] Adams GL, Gansky SA, Miller AJ, Harrell WE, Hatcher DC (2004). Comparison between traditional 2-dimensional cephalometry and a 3-dimensional approach on human dry skulls. Am J Orthod Dentofac Orthop.

[17] Halazonetis DJ (2005). From 2-dimensional cephalograms to 3-dimensional computed tomography scans. Am J Orthod Dentofac Orthop.

[18] Grauer D, Cevidanes LS, Proffit WR (2009). Working with DICOM craniofacial images. Am J Orthod Dentofac Orthop.

[19] Terajima M, Yanagita N, Ozeki K, Hoshino Y, Mori N, Goto TK (2008). Three dimensional analysis system for orthognathic surgery patients with jaw deformities. Am J Orthod Dentofac Orthop.

[20] Park JH, Tai K, Owtad P (2015). 3-dimensional cone-beam computed tomography superimposition: a review. Semin Orthod.

[21] Steuer I (1972). The cranial base for superimposition of lateral cephalometric radiographs. Am J Orthod.

[22] Cevidanes LH, Heymann G, Cornelis MA, DeClerck HJ, Tulloch JF (2009). Superimposition of 3-dimensional cone-beam computed tomography models of growing patients. Am J Orthod Dentofac Orthop.

[23] Gkantidis N, Schauseil M, Pazera P, Zorkun B, Katsaros C, Ludwig B (2015). Evaluation of 3-dimensional superimposition techniques on various skeletal structures of the head using surface models. PLoS One.

[24] Lee JH, Kim MJ, Kim SM, Kwon OH, Kim YK (2012). The 3D CT superimposition method using image fusion based on the maximum mutual information algorithm for the assessment of oral and maxillofacial surgery treatment results. Oral Surg Oral Med Oral Pathol Oral Radiol.

[25] Park SB, Yoon JK, Kim YI, Hwang DS, Cho BH, SOn WS (2012). The evaluation of the nasal morphologic changes after bimaxillary surgery in skeletal class III maloccusion by using the superimposition of cone-beam computed tomography (CBCT) volumes. J Craniomaxillofac Surg.

[26] Cevidanes LH, Bailey LJ, Tucker SF, Styner MA, Mol A, Phillips CL (2007). Three-dimensional cone-beam computed tomography for assessment of mandibular changes after orthognathic surgery. Am J Orthod Dentofac Orthop.

[27] de Oliveira AE, Cevidanes LH, Phillips C, Motta A, Burke B, Tyndall D (2009). Observer reliability of three-dimensional cephalometric landmark identification on cone-beam computerized tomography. Oral Surg Oral Med Oral Pathol Oral Radiol Endod.

[28] Hwang JJ, Kim KD, Park H, Park CS, Jeong HG (2014). Factors influencing superimposition error of 3D cephalometric landmarks by plane orientation method using 4 reference points: 4 point superimposition error regression model. PLoS One.

[29] Lagravère MO, Secanell M, Major PW, Carey JP (2011). Optimization analysis for plane orientation in 3-dimensional cephalometric analysis of serial cone-beam computerized tomography images. Oral Surg Oral Med Oral Pathol Oral Radiol Endod.

[30] Ruellas A, Huanca L, Gomes MT (2015). Comparison and Reproducibility of two maxillary regional registration methods. J Dent Res.

[31] Ruellas AC, Tonello C, Gomes LR, Yatabe MS, Macron L, Lopinto J (2016). Common 3-dimensional coordinate system for assessment of directional changes. Am J Orthod Dentofac Orthop.

[32] Lagravère MO, Major PW (2005). Proposed reference point for 3-dimensional cephalometric analysis with cone-beam computerized tomography. Am J Orthod Dentofac Orthop.

[33] Lagravère MO, Gordon JM, Flores-Mir C, Carey J, Heo G, Major PW (2011). Cranial base foramen location accuracy and reliability in cone-beam computerized tomography. Am J Orthod Dentofac Orthop.

[34] Lagravère M, Major P, Carey J (2010). Sensitivity analysis for plane orientation in three-dimensional cephalometric analysis based on superimposition of serial cone beam computed tomography images. Dentomaxillofac Radiol.

[35] Nada RM, Maal TJ, Breuning KH, Bergé SJ, Mostafa YA, Kuijpers-Jagtman AM (2011). Accuracy and reproducibility of voxel based superimposition of cone beam computed tomography models on the anterior cranial base and the zygomatic arches. PLoS One.

[36] Nguyen T, Cevidanes L, George W (2014). Validation of 3D mandibular regional superimposition methods for growing patients (Abstract). J Dent Res.

[37] Björk A (1955). Cranial base development: a follow-up x-ray study of the individual variation in growth occurring between the ages of 12 and 20 years and its relation to brain case and face development. Am J Orthod.

[38] Currie K (2017). Posterior cranial base growth and development changes as assessed through CBCT imaging in adolescents [dissertation].

[39] Proffit W, Fields HW, Sarver DM. Chapter 2: concepts of growth and development in contemporary orthodontics: Mosby; 2012.

[40] Van der Linden F. Facial growth and facial orthopedics: quintessence; 1989.

[41] Enlow DH, Harris DB (1964). A study of the postnatal growth of the human mandible. Am J Orthod.

[42] Enlow DH. Essentials of Facial Growth. 2nd ed: Needham Press; 2008.

[43] Carlson DS, Buschang PH. Chapter 1: craniofacial growth and development: developing a perspective in orthodontics: current principles and techniques. Graber, LW; Vanarsdall RL; Vig KW; Huang GJ. Sixth edition ed. St. Louis, Missouri: Elsevier Health Sciences. Kindle Edition; 2017.

[44] Björk A, Skieller V (1977). Growth of the maxilla in three dimensions as revealed radiographically by the implant method. Br J Orthod.

[45] Buschang PH, Julien K, Sachdeva R, Demirjian A (1992). Childhood and pubertal growth changes of the human symphysis. Angle Orthod..

[46] Loubele M, Jacobs R, Maes F, Denis K, White S, Coudyzer W (2008). Image quality vs radiation dose of four cone beam computed tomography scanners. Dentomaxillofac Radiol..

[47] Damstra J, Fourie Z, Huddleston Slater JJ, Ren Y (2011). Reliability and the smallest detectable difference of measurements on 3-dimensional cone-beam computed tomography images. Am J Orthod Dentofac Orthop.

